# Role of Phospholipid Flux during Milk Secretion in the Mammary Gland

**DOI:** 10.1007/s10911-017-9376-9

**Published:** 2017-02-27

**Authors:** Michał Smoczyński

**Affiliations:** Department of Dairy Science and Quality Management, Faculty of Food Science, University of Warmia and Mazury in Olsztyn, Oczapowskiego Str. 7, 10-719 Olsztyn, Poland

**Keywords:** Phospholipid flux, Milk lipids, Mammary gland, Milk secretion

## Abstract

Lipids are a complex group of chemical compounds that are a significant component of the human diet and are one of the main constituents of milk. In mammals, lipids are produced in the milk-secreting cells in the form of milk fat globules. The chemical properties of these compounds necessitate developing separate processes for effective management of non-polar substances in the polar environment of the cell, not only during their biosynthesis and accumulation in the cell interior and secretion of intracytoplasmic lipid droplets outside the cell, but also during digestion in the offspring. Phospholipids play an important role in these processes. Their characteristic properties make them indispensable for the secretion of milk fat as well as other milk components. This review investigates how these processes depend on the coordinated flux and availability of phospholipids and how the relationship between the surface area (phospholipids) and volume (neutral lipids) of the cytoplasmic lipid droplets must be in biosynthetic balance. The structure formed as a result (i.e. a milk fat globule) is therefore a result of specified structural limitations inside the cell, whose overcoming enables the coordinated secretion of milk components. This structure and its composition also reflects the nutritional demands of the developing infant organism as a result of evolutionary adaptation.

## Introduction

An increasing number of studies have demonstrated that breastfeeding is one of the key factors affecting the health of infants and has a positive impact on mothers by minimizing the likelihood of breast and ovarian cancer [1, 2]. This synergistic, positive effect on both the baby and the mother results from evolutionary adaptation and development of mechanisms indispensable for ensuring the best possible conditions for progeny development.

One of the constituents of milk are lipid compounds, which are an important component of the human diet throughout the whole life. Lipids are a complex group of chemical compounds, the significance and role of which are probably the least recognized among all groups of biomolecules. Due to their complex composition and incomplete knowledge in this regard, it is difficult to define the nutritional recommendations concerning this group of compounds, even though they constitute a significant part of the cell metabolome. The reason for this may be slower progression in available instrumentation to adequately characterize complex lipid fingerprints. Genetic and bioinformatic methods are not very useful in the analysis of lipids since they are not directly linked with the body genome. Therefore, progress in this research area is proceeding slightly slower than other classes of biomolecules [3]. Although lipid diagnostics has long been applied in medicine (blood levels of cholesterol, lipoproteins or triacylglycerols), the structural foundations of the role of qualitative composition of these compounds remain largely unexplained. Although our knowledge of multiple lipid roles is increasing, more research is needed to understand the unique attributes of structurally specific lipid species in relation to cell function. This indicates the necessity for the development of this research area, all the more that one of the basic functions of lipids is providing and accumulating energy and that an excess of energy reserves in the human body poses one of the major nutritional problems facing the contemporary generation.

Milk lipids in the form of milk fat globules (MFG) are produced in the milk-secreting cells, a single layer of which lines the internal walls of mammary gland cavities, called alveoli. The diversity of synthesized lipids and complexity of biosynthetic mechanisms in the mammary gland point to multiple potential roles, to ensure the appropriate growth, health and survival of the progeny of mammals and, at the same time, reflect structural determinants stemming from the chemical properties of these compounds. This has required adapting separate processes for effective management of non-polar substances in the polar environment of the cell, not only during their biosynthesis and accumulation in the cell interior and secretion of intracytoplasmic lipid droplets outside the cell, but also during digestion in a progeny’s body. These processes have to be strictly coordinated and phospholipids play an important role in these processes.

The digestion of lipid substances proceeds along with digestion of other dietary components. As a result of complex physicochemical mechanisms, this leads to the transformation of non-polar compounds into more polar components, which are capable of overcoming the unstirred layer of fluid on the surface of epithelial cells of the intestine and enables their absorption by villi [4]. Hence, the structure and properties of lipid in milk is strictly related to the processes of their digestion and have evolved together, adjusting to each other in order to maximize the effectiveness of both processes. An example of such an adjustment may be the high degree of milk fat dispersion which results from mechanisms leading to the formation of fat globules as well as through increasing the interface, which allows for more efficient access of lipase to a lipid globule interior and more effective degradation of the substrate, i.e. triacylglycerols present inside lipid droplets.

Our knowledge of the properties, composition and occurrence of particular groups of lipid compounds is expanding due to the application of such analytical methods as chromatography, mass spectrometry (MS) and nuclear magnetic resonance (NMR) spectroscopy. Complete databases of lipid compounds typical of particular tissues have also been established [5, 6]. This unique multiplicity of structures and their diversified composition also indicate the variety of functions they play and their complex roles in a living organism. Only determination of the correlations between their composition and biological properties will allow exploiting this knowledge to improve the quality of foods and prevent and treat many different diseases.

This review investigates the properties of phospholipid compounds occurring in milk and their effect on the structure of a milk fat globule and identifies their potential role during milk secretion in the mammary gland.

## Composition and Structure of Milk Lipids

### Lipids and Proteins of the Milk Fat Globule

The content of lipid compounds in milk is species-dependent and ranges from 3.8 to 3.9% in human milk and from 3 to 5% in cow’s milk. They occur in the form of the so-called milk fat globules with sizes ranging from 0.2–15 μm. Inside the globules, there are triacylglycerols which represent 97–98% of milk lipids [7]. They are coated with a triple phospholipid-protein layer which is 10–20 nm in diameter, the so-called milk fat globule membrane (MFGM). This triple membrane is a unique structure in biology; among all known cells, only the epithelial cells of the mammary gland are capable of secreting intracytoplasmic lipid droplets outside a cell [8]. The membrane of MFG constitutes 2–6% of the total globule weight and is composed mainly of proteins and phospholipid, which represents at least 90% of the membrane. The content of proteins in the membrane is estimated at 22–60% [9–11], about 1–2% of the total protein weight in milk. The main proteins of the milk fat globule membrane are presented in Table 1.

**Table 1:** The main protein components of the bovine milk fat globule membrane (adapted from Ref. [12])

Proteins (other names or abbreviations)	Molecular weight, (Da)
Mucin 1 (MUC 1)	120,000–220,000
Xanthine dehydrogenase/oxidase (XDH/XO)	300,000 (2 × 150,000)
Periodic acid Schiff III (PAS III, Mucin 15, MUC 15)	95,000–100,000
CD 36 (PAS IV)	76,000–78,000
Butyrophilin (BTN)	66,000–67,000
Periodic acid Schiff (PAS VI/VII, lactadherin, milk fat globule-EGF factor 8)	43,000–59,000
Adipophilin (ADPH)	52,000
Fatty acid binding protein (FABP)	13,000

Some of the membrane proteins are glycoproteins and can be covalently linked with phospholipids or neutral lipids [9]. Apart from these major proteins, membrane preparations also contain many other proteins, such as: enzymes, immunoglobulins, MHC proteins and proteins derived from the cytoplasm of epithelial cells of the mammary gland and leucocytes [12, 13]. The lipid composition of the milk fat globule membrane is presented in Table 2.

**Table 2:** Lipid composition of the bovine milk fat globule membrane (adapted from Ref. [11, 14])

Lipid component	% of total lipids of the MFGM *- % of phospholipids
Triacylglycerols	56.0–62.0
Diacylglycerols	2.1–9.0
Monoacylglycerols	0.4
Free fatty acids	0.6–6.0
Sterols	0.2–2.0
Sterol esters	0.1–0.3
Phospholipids	26.0–40.6
Phosphatidylcholine (PC)	*31.0–36.0
Phosphatidylethanolamine (PE)	*27.0–30.5
Sphingomyelin (SM)	*19.9–22.0
Phosphatidylinositol (PI)	*7.1–11.0
Phosphatidylserine (PS)	*4.0–5.0
Lactosyl-cerebroside	*3.4
Lysophosphatidylcholine	*2.0
Glucosyl-cerebroside	*0.3

Although the lipid of the milk fat globule membrane comprises both neutral and polar lipids, however, the triacylglycerol fraction may originate from contamination by the core of fat globule during isolation of the membrane [15]. The content of di-, monoacylglycerols and free fatty acids may also result from the enzymatic activities during preparation of the sample.

The main polar lipids of milk consist of phospho- and sphingolipids. They are basic components of the membranes due to their amphiphilic structure. The first are built of glycerol esterified at two sites with fatty acids (FA) and with phosphate with a different organic group (e.g. serine, choline) attached at the third site in the glycerol backbone. For sphingolipids, the structure varies and is based on a sphingosine (the sphingoid base), i.e. a long-chain aliphatic amine which additionally contains two or three hydroxyl groups. The attachment of a fatty acid to an amine group results in the formation of ceramide. After the addition of the organophosphate group, a sphingophospholipid is formed. Once phosphocholine is attached to the ceramide, sphingomyelin is formed, whereas a sphingoglycolipid is formed upon the attachment of a saccharide group [16, 17]. Phospholipids constitute ca. 1% of milk fat [18] and most of them are formed within the endoplasmic reticulum (ER) [19].

### Structural Features of the Milk Fat Globules

The structure of a fat globule membrane is asymmetric – the internal and external surfaces differ from each other with their composition, which is due to the wide range of functions served by a given surface. The components of the MFGM are arranged so that the external part of the bilayer is constituted mainly by phospholipids containing choline (PC and SM) as well as glycolipids, cerebrosides, and gangliosides (molecules containing polar components as saccharide residues), directed towards the aqueous environment, whereas the interior surface of the bilayer contains phosphatidylethanolamine, phosphatidylinositol and phosphatidylserine [9, 20].

Structurally, the MFGM is a so-called liquid lipid-protein mosaic. According to the widely-accepted current model of the membrane, the so-called “liquid lipid-protein mosaic”, proteins are immersed or flow in a liquid bilayer of spatially-oriented lipids [21]. Membrane components (both proteins and phospholipids) are capable of lateral diffusion if not coupled with components of the cytoskeleton of a cell. For phospholipids, diffusion across the membrane is also likely. In the bilayer, phospholipids may enter into specific interactions with proteins, thereby affecting their biological properties and functions. Apart from that, local areas with a composition and properties differing from other areas with incidental composition may also occur in the membrane. These are so-called “lipid rafts” which may be isolated from the membrane with appropriately selected solvents [22]. Agglomerations of sphingolipids with cholesterol may serve as an example in this case. Sphingolipids display some typical traits that distinguish them from other phospholipids. They contain relatively more long-chain and saturated fatty acids, which allows for dense packing of the hydrocarbon chains next to one another. In addition, due to structural matching, they show affinity to cholesterol and may form lateral bonds with it, based on both polar and non-polar interactions. For this reason, these domains do not occur in a liquid state, unlike the remaining part of the membrane. As the lateral diffusion of components of these domains is still likely, it is not a solid state either (below melting point). It is assumed that they occur in an intermediate state, the so-called liquid ordered phase (Lo) [23]. Long chain n-3 polyunsaturated fatty acids (PUFA) can modify the molecular order within cholesterol-enriched lipid microdomains. Recent studies have shown that eicosapentaenoic acid (EPA) and docosahexaenoic acid (DHA) upon incorporation into the plasma membrane exert different structural effects when incorporated into phosphatidylethanolamines (PE) compared to phosphatidylcholines (PC). DHA-containing PE moves into a non-raft region and push cholesterol into the rafts and increase molecular order within the membrane, while DHA-containing PC locate into the rafts, displace cholesterol from rafts and the molecular order in membrane decreases [24]. Proteins and glycolipids may also occur in the rafts or on their rims. The grouping of components with similar properties is consistent with the principle of energy minimization. Regarding their functional properties, they may take part in many vital processes of a cell involving signal transmission or inter-cell communication. The action of several anticarcinogenic drugs is based on the induction of cancer cell apoptosis through disruption of the integral structure of lipid rafts in their membrane [25]. The presence of lipid rafts was also observed in MFG [26], although their role and function in nutrition remains unexplained.

Considering the structure itself and structure-related physicochemical properties, the role of phospholipids includes stabilization of milk fat in the dispersed form, prevention of flocculation and coalescence of globules, as well as protection against adverse effects of lipases. Apart from their structural functions in the membrane, phospholipids are also significant in nutrition in the period of infant development. Several studies have demonstrated the high biological activity of these compounds and their metabolites, which allows classifying them as components with a positive effect upon human health [20]. Their biological properties have been widely discussed in review articles [27–29]. For example, treatment with phosphatidylserine-containing liposomes was shown to positively influence patients suffering from Alzheimer’s disease [30]. Sphingomyelin was shown to present potentially anti-cancer properties [31]. Other examples include the antitoxic properties of phosphatidylcholine [32] and the positive effect on the development and functioning of the nervous system [33], as well as the anti-bacterial properties of phospholipid and triacylglycerols hydrolysis products [34]. Also phospholipids containing (n-3) fatty acids may have protective properties against obesity in childhood [35].

## Structural Determinants of Milk Fat Globules Secretion – the Role of Phospholipids

### Basic Properties of Polar Lipids

In terms of chemical structure, molecules present in the world around us may be divided into those which possess a charge and those which have no charge in their structure. The possession of charge results in specified properties that are referred to as hydrophilic and lipophilic, respectively. Phospholipids merge these two properties and constitute a structural hybrid composed of a part which possesses a charge and a part with no charge, the so-called “hydrophilic head and hydrophobic tail”. These are referred to as amphipathic compounds. This structure determines the specific properties of phospholipids. As each system tends to form the lowest energy state, the most beneficial would be if the hydrophilic part could contact the hydrophilic environment and avoid the hydrophobic environment. By analogy, contact of the hydrophobic part with the hydrophilic environment would be energetically unbeneficial. In an aqueous environment, phospholipid orientation enables the hydrophilic part to contact water and simultaneously the hydrophobic part to hide from contact with water. Such conditions are met when phospholipids are at the water surface and when their hydrophilic part is directed towards water. In this case, the surface tension of the water decreases. Another potential arrangement is a lipid bilayer in which the hydrophobic parts are hidden from contact with water inside the bilayer. If such a bilayer binds into a spherical structure, a liposome will form that separates the two spaces. This ability has resulted in the possibility of developing a structure separated from the external environment, i.e. a cell with a separating bilayer called a biological membrane. Considering that one tip of the phospholipids is polar and the other is non-polar, an ideal situation would be the hydrophilic part having contact with the hydrophilic environment and the hydrophobic part having contact with the hydrophobic environment. This is likely when the phospholipid is at the contact site of these two environments, i.e. at the so-called interface. Another property of phospholipids stemming from their structure is their emulsifying capability, which consists in the ability to disperse the non-polar molecules in the polar environment in a dispersed form (or otherwise, i.e. dispersing polar molecules in a non-polar environment).

Although the general structure of phospholipids is similar, the significant variations in their chemical make-up in biological membranes entail some differences in their properties. These properties depend on both the length and saturation of attached fatty acids and the type and polarity of the head group. Different studies of these properties in artificial membrane systems indicate that the chain length, degree of unsaturation and properties of the polar head group and the type of pairing of the hydrocarbon chains may affect the rate of diffusion and transport through the membrane [36]. Phospholipid non-polar tails associate with the involvement of van der Waals hydrophobic interactions, which is of great importance for the integrity of the biological membranes. These interactions increase with an increase in the acyl chain length, but decrease with an increase in the number of unsaturated bonds in the hydrocarbon chain. Double bonds in the hydrocarbon chains introduce a kink which disrupts part of the hydrophobic interactions and increases the fluidity of the membrane. The double bond in membrane phospholipids is usually in the cis configuration. Polar head groups contact with water environment and determine the net surface charge of the membrane. Electrostatic forces may also play a role in stabilizing the integrity of the membrane, but may also allow for binding other polar lipids or proteins.

These general basic examples show how the specified properties and characteristics of phospholipids determine their behaviour in various environments. They also point to some limitations a cell has to overcome when using non-polar substances in a polar environment as well as to the specific role of phospholipids in these processes.

### Regulation of Milk Fat Biosynthesis

Processes related to lipid metabolism and leading to the biosynthesis of milk fat include a number of steps. One of the main constituents of milk triacylglycerols and phospholipids are fatty acids. They may come from ingested food or maternal fat stores. In addition, some fatty acids (saturated mainly of 10–14 carbon atoms) may be synthesized de novo in the liver, mammary gland and other tissues [37]. Fatty acids from ingested food after absorption by the intestinal villi in the small intestine, undergo re-esterification into triacylglycerol and in the form of chylomicrons are delivered to the mammary gland [38]. During periods of a shortage of dietary fat, triacylglycerols are transported from the liver to the mammary gland in the form of VLDL (very low density lipoproteins). Fatty acids may also be transported in the body with albumin [40] The main protein component of both chylomicrons and VLDL is apolipoprotein B, which interacts with lipoprotein receptors on the surface of mammary cells [39]. Fatty acids are then released under the influence of lipoprotein lipase (LPL) and may cross the membrane and enter the cell [40]. During lactation, the level of LPL is increased in the mammary gland and decreased in adipose tissue, indicating increased utilization of fatty acids in the mammary gland [41]. CD36 protein may play an important role in the import of fatty acids into the cell [42].

**Fig. 1:**
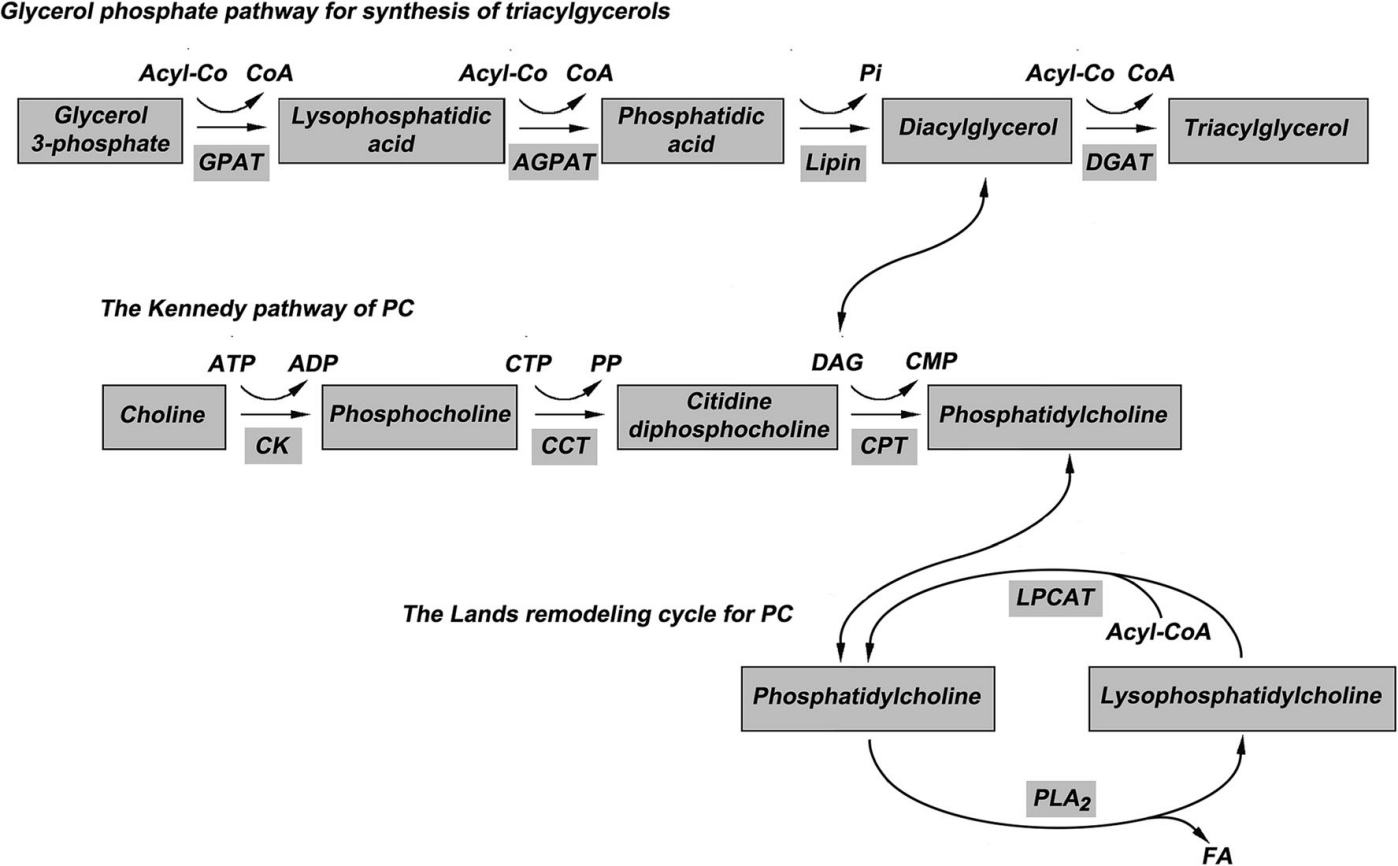
General scheme for non-polar and polar lipid biosynthesis (based on Ref. [43–45]). Glycerol phosphate pathway for synthesis of triacylglycerols. Two subsequent acylation steps of glycerol phosphate by glycerol 3-phosphate acyltransferase (GPAT) and 1-acylglycerol-3-phosphate acyltransferase (AGPAT) lead to phosphatidic acid (PA). A lipin family enzyme then converts PA into diacylglycerol (DAG). DAG can enter the Kennedy pathway for phosphatidylcholine or after the addition of a third acyl chain can be converted to triacylglycerol (TAG) through the action of diacylglycerol acyltransferase (DGAT). The Kennedy pathway of PC. After phosphorylation of choline catalysed by choline kinase (CK) CTP:phosphocholine cytidylyltransferase (CCT) catalyses activation of phosphocholine with cytidine triphosphate (CTP). This activated form is transferred to DAG and PC is formed. This reaction is catalysed by cholinephosphotransferase (CPT). The remodeling Lands cycle for PC. Phospholipase A2 (PLA2) hydrolyses the acyl chain from the sn-2 position of a PC and generates a lysophosphatidylcholine (LPC). Subsequently lysophosphatidylcholine acyltransferase (LPCAT) catalyses reacylation of LPC with a second fatty acid (in the form of fatty acyl-CoA). Other abbreviations: ATP-adenosine triphosphate; ADP-adenosine diphosphate; CDP-cytidine diphosphate; CMP-cytidine monophosphate; PP-diphosphate; CoA-coenzyme A

After crossing the membrane, fatty acids under the influence of acyl-CoA synthetase are activated into the form of an acyl CoA. The activated fatty acids connected to specific cytosolic FABP (fatty acid binding protein) are transported to intracellular organelles for further reactions. Depending on the needs of the cells, fatty acids can be desaturated under the influence of stearoyl-CoA desaturase (SCD) and/or be converted into triglycerides, phospholipids or cholesterol esters [42]. Glycerol phosphate acyltransferase (GPAT), acylglycerolphosphate acyltransferase (AGPAT), lipin (phosphatidate phosphatase) and diacylglycerol acyltransferase (DGAT) are responsible for the conversion of glycerol-3-phosphate to lysophosphatidic acid, phosphatidic acid, di- and triacylglycerol, respectively, and are present in the membrane of the endoplasmic reticulum. Figure 1 summarizes these reactions, which lead to biosynthesis of triacylglycerols as well as the Kennedy CDP-choline pathway and Lands` remodeling cycle, leading to phosphatidylcholine biosynthesis [43–45].

The biosynthesis of milk fat is a complex process which requires coordinated control of many cellular processes and metabolic pathways occurring at different stages of development and functioning of the mammary gland. These processes are regulated by the so-called gene networks, which are interactions and relationships between transcription factors and other regulatory elements that affect the expression of specific genes [42, 46]. During each pregnancy changes in hormone levels induce cycles of growth, lactation and involution, during which mammary epithelial cells proliferate and differentiate to produce milk and then die in the apoptotic process [47]. Prolactin and oxytocin are essential hormones for the production and secretion of milk during the lactation period [48, 49]. Binding of prolactin to its receptor (PRLR) on the surface of cells of the mammary gland results in its dimerization. Dimerization leads to activation of the Janus tyrosine kinase, receptor phosphorylation and dimerization and phosphorylation of Stat transcription factors Stat 5a and 5b. In this form, these factors can move into the nucleus and, by the addition to the appropriate regulatory DNA sequences, activate milk proteins genes [50]. Naylor et al. [51] used transcript profiling of three mouse models that exhibit failure of prolactin secretory activation and observed changes in gene expression. They found reduction in expression of many genes representing milk proteins, metabolism, immune response, key transcription factors and also lipid, cholesterol and fatty acid biosynthetic enzymes.

Proteins belonging to the family of SREBP proteins are transcription factors binding sterol regulatory element (sterol regulatory element-binding proteins, SREBP). They regulate the expression of genes involved in cholesterol and lipid homeostasis. Their regulatory domain interacts with the SCAP protein (SREBP cleavage activating protein) which acts as a sensor responsive to the level of cholesterol. When the cholesterol level is lowered, the protein SCAP escorts SREBP protein to the Golgi apparatus where, under the influence of two specific proteases, the domain that interacts with DNA is released. This fragment after dimerization translocates to the nucleus and, when attached to the promoter regions of DNA, activates the entire group of genes responsible for the synthesis of cholesterol, fatty acids, triglycerides, phospholipids and NADPH (as a cofactor necessary for the synthesis of these compounds) [52]. Rudolph et al. [53] studied role of sterol regulatory element binding proteins in regulation of fatty acid synthesis using mouse models with mammary epithelial cell-specific deletion of SCAP and SREBF1 isoforms. They identified some target genes of SCAP and SREBF proteins, but the obtained results also suggest a role for additional regulatory mechanism for de novo fatty acid synthesis in the mammary gland.

Another regulator of lipid metabolism in the mammary gland may be a PPAR γ protein (peroxisome proliferator-activated receptor γ). This protein is involved in the process of adipocyte differentiation and its expression can be stimulated by either insulin or SREBP-1. It belongs to the transcription factors activated by lipophilic agents and regulates the expression of genes related to the metabolism of fats, proteins and carbohydrates [54]. It has been shown that the activity of many enzymes (of their various isoforms) is governed largely by complexes of fatty acids with FABP (fatty acid binding protein) which determines their further utilization [55]. These complexes can also affect gene expression by interacting with the PPAR γ [42].

While the relationship between transcription factors regulating the biosynthesis of milk fat has not been precisely defined, many important elements playing a primary role in this regulation have been identified. Most of research concentrated on the regulation of fatty acid or triacylglycerol synthesis. For example Cases et al. [56] showed that mice lacking the triglyceride synthesis enzyme acyl CoA:diacylglycerol transferase 1 (DGAT) are characterized by impaired mammary gland development. Contrary, not much research concentrated on the role of phospholipids biosynthesis regulation in the mammary gland functioning. Doria et al. [57] used genomic and lipidomic approaches to characterize lipid metabolism changes in mammary epithelial cells undergoing proliferation and differentiation and study correlation to breast cancer survival. They showed that many genes involved in phospholipid synthesis and metabolism were upregulated in differentiating mammary cells and also correlated with breast cancer survival.

This brief overview of some elements relating to the regulation of milk fat biosynthesis indicates the need for further research and greater attention to the role of phospholipid biosynthesis in the functioning of the mammary gland as well as in relation to the overall metabolism of lipids.

### Formation of Intracellular Lipid Droplets

The formation of lipid droplets in the cytoplasm is linked with endoplasmic reticulum. Also phospholipid synthesis occurs in the cytosol in close proximity to the ER membrane. After incorporation into the membrane, fragments of the membrane can eventually bud off and transport phospholipids across the cell. Triacylglycerols accumulate on the surface or inside the ER membrane. By forming a “bulge”, they may detach from the ER membrane and migrate to the cytoplasm and become surrounded by the outer cytosolic part of the ER membrane which contains polar lipids and proteins [58, 59]. This monolayer separates the lipid core of a lipid droplet from the cell’s interior. Adipophilin (ADPH) belongs to the PAT proteins (perilipin, adipophilin and TIP47) family and is known to specifically localize to cytoplasmic lipid droplet surface and regulate the access to the droplet interior in mammary gland [60]. In the cytoplasm, the small droplets fuse with each other, forming droplets of various larger sizes. Although the mechanisms that regulate this process are still unclear, it has been demonstrated that small droplets may merge into larger droplets, whereas the larger ones are incapable of fusion [8]. The SNAP (soluble NSF attachment protein) and SNARE (soluble N-ethylmaleimide-sensitive-factor (NSF)-attachment protein receptor) proteins may participate in the fusion process of intracytoplasmic lipid droplets [61]. Other proteins may be also linked to the size of lipid droplets [62]. Cell death-inducing DFF45-like effector (CIDE) family proteins have recently gained attention as key regulators of lipid storage [63]. It was shown that cells deficient in Fsp27 (fat specific protein of 27 kDa) or Cidea, members of CIDE family, accumulate many small intracellular lipid droplets [64]. The FITM (fat storage inducing transmembrane) proteins are evolutionarily-conserved membrane proteins. The breakdown of these proteins leads to reduced storage of triacylglycerols [65], while their overexpression significantly increases TAG storage and droplet size [66]. Perilipin 1 (another PAT protein) is highly expressed in adipocytes, where it locates on the surface of lipid droplets and is a well-established regulator of lipolysis in adipocytes [67]. These proteins are known regulators of processes related to the functioning of lipid droplets, but little is known about the role of phospholipids in these processes and possible interactions between proteins and lipids at the interface.

Afterwards, by means of an unknown mechanism, the droplets are transported to the apical part of a secretory cell membrane. Studies have demonstrated high contents of microtubules, actin microfilaments and intermediate filaments in the apical regions of secretory cells [68, 69], as well as the presence of proteins interacting with the cytoskeleton in the intracellular lipid droplets [70]. The involvement of cytoskeleton elements in the intracellular transport of lipid droplets therefore seems likely.

### Crossing the Plasma Membrane - the Phospholipid Flux is Essential for Continuity of Milk Component Secretion

Fat globules are intracytoplasmic lipid droplets secreted outside the milk-producing cells. These are the only known cells capable of secreting whole lipid droplets outside the cell. It has required “developing” a separate mechanism enabling this process.

The lipid droplets are secreted outside the cell, probably according to a unique mechanism different from classical exocytosis. This mechanism differs completely from the secretion of triacylglycerols by other cells (e.g. secretion of chylomicrons or other lipoproteins), although two potential mechanisms have been postulated.

**Fig. 2:**
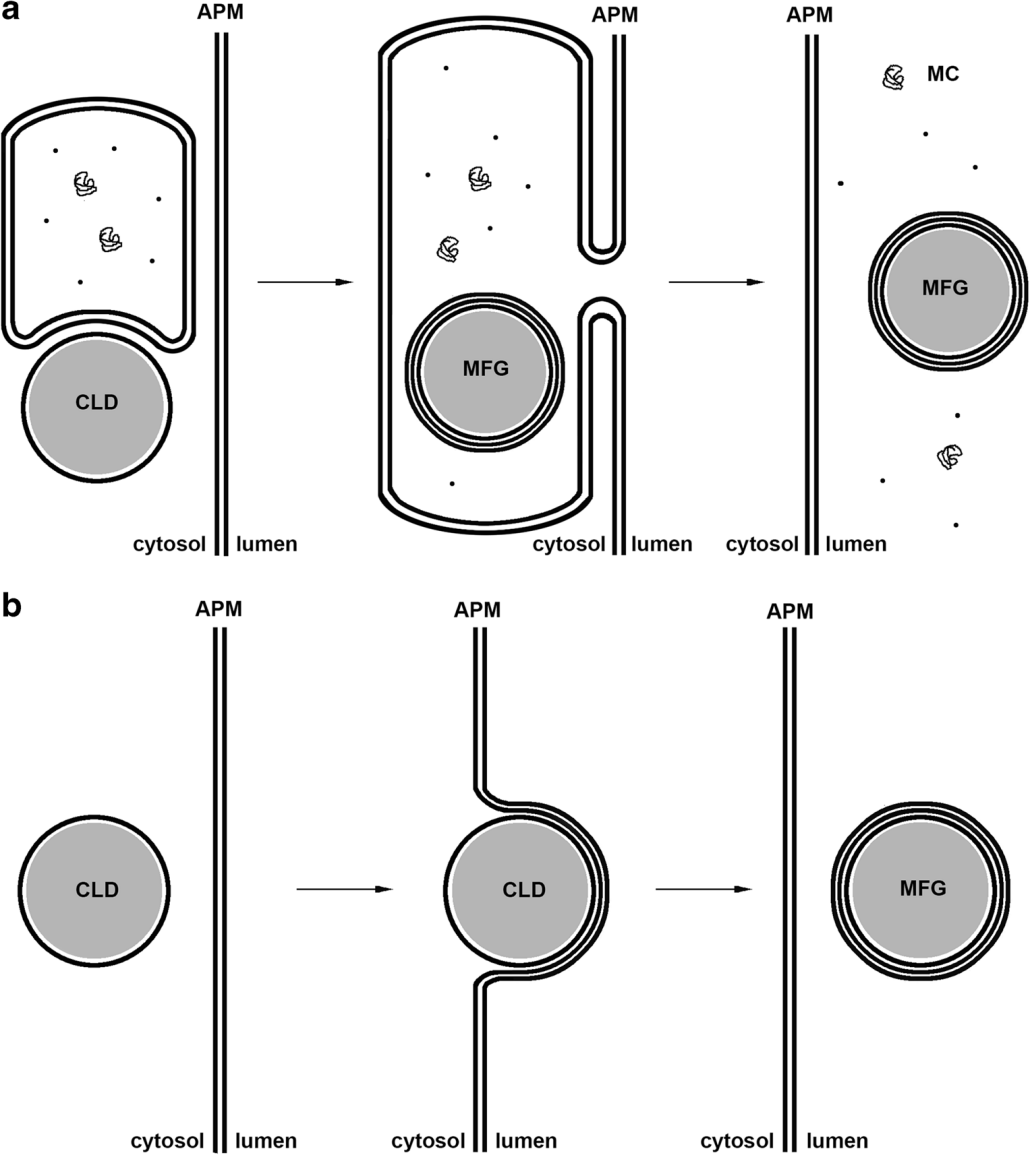
Theoretically possible schemes of milk fat globule secretion. In Fig. 2a, the classical model of secretion of milk components with the formation of an intracytoplasmic vacuole containing both casein micelles and lipid droplets. The content of this vacuole is then secreted outside the cell via exocytosis. Fig 2b presents the modern, currently accepted model of secretion of milk components. In this model intracellular lipid droplets contact directly with the apical plasma membrane and are secreted outside the cell surrounded by a triple layer of phospholipids. Considering the flux of phospholipid, only the scheme presented in Fig. 2b seems plausible. Abbreviations: APM-apical plasma membrane; CLD-cytoplasmic lipid droplet; MFG-milk fat globule; MC-milk components

In the classical model, alveoli are subject to fusion, which leads to the formation of an intracytoplasmic vacuole containing both casein micelles and lipid droplets. The contents of these vacuoles are then secreted outside the cell via classical exocytosis [71]. A potential scheme of such a mechanism considering the flux of phospholipids is presented in Fig. 2a.

The second, currently accepted potential mechanism assumes that lipid droplets are coated with a cellular membrane originating from the external, apical part of the secretory cells and, in this way, gain an additional lipid bilayer [72]. This bilayer contains multiple membrane proteins, some of which take part in the secretion of lipid droplets. A significant role may be ascribed in this case to butyrophilin and xanthine dehydrogenase/oxidase (XDH). These proteins have been found to interact with each other during secretion of lipid droplets because they occur in stable molar ratios in a membrane of lipid droplets, among others [8]. As butyrophilin is linked with a cell membrane and XDH is a cytosolic enzyme, interactions may occur between the cytosol part of butyrophilin and XDH during lipid droplet secretion, which results in the formation of complexes on the droplet surface with disulfide bridges. Adipophilin (ADPH) may also participate in these interactions and in the formation of a protein coat on a droplet surface [8, 73]. These complexes may initiate the process of droplet coating with a membrane and its secretion outside the cell (Fig. 2b).

**Fig. 3:**
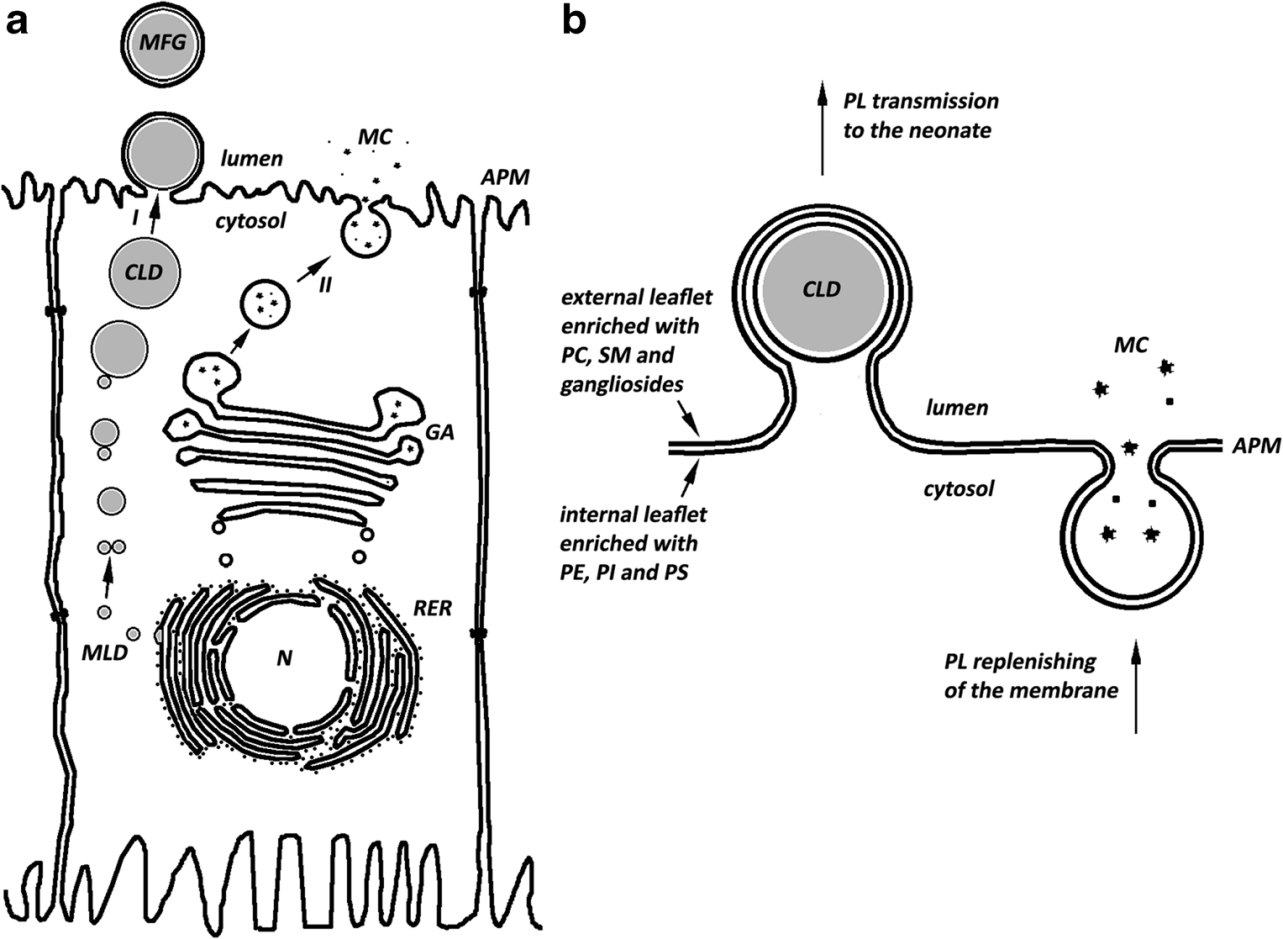
Scheme of secretion of milk components (Fig. 3a – based on Ref. [72]). In Fig. 3b, arrows indicate the flux of phospholipids to and from the apical membrane of the mammary gland. In this model milk fat globules and water soluble milk components are secreted separately, by two distinct pathways (I and II, respectively). These two pathways enable replenishing the phospholipid content of the apical membrane, maintaining its constant composition and, thereby, the continuous secretion of milk components. Abbreviations: MFG-milk fat globule, CLD-cytoplasmic lipid droplet, MLD-microlipid droplet, N-nucleus, MC-milk components, APM-apical plasma membrane, GA-Golgi apparatus, RER-rough endoplasmic reticulum, PL-phospholipid

This mechanism results in the formation of a specific structure and, although this unique structure results from the mechanism of lipid droplet secretion outside the cell, this mechanism is also expected to ensure specified benefits to the progeny. It is known that the lipid droplets secretion process probably follows the mechanisms presented in Fig. 2b. In this process, the droplet is coated by the cellular membrane and after membrane fusion it is secreted as a droplet coated with a triple lipid layer [72]. This mechanism may result from some limitations, but it may also yield some benefits to the progeny. Above all, it ensures the flux of phospholipids in the cell secreting lipid droplets outside the cell which, in turn, allows for effective secretion of all milk components, as depicted in Fig. 3a. On the one hand, the vesicles provide water-soluble substances in the form of transporting liposomes coated with a double lipid layer. Proteins synthesized in a rough endoplasmic reticulum (RER) are secreted in this way. After translation and penetration into the interior of reticulum, the primary structures of proteins emerge in the transporting vesicles and are transported to the Golgi apparatus, from where (after modifications and development of the specific protein structure) they migrate again in transporting vesicles to the apical part of the cellular membrane to be eventually secreted outside the cell. The contents of these structures are secreted according to the mechanism of classical exocytosis, after the fusion of the transporting vesicle with the cellular membrane [74]. In this case, the membrane material is delivered with the vesicles and remains in the apical part of the cellular membrane. In this way, the membrane material would shortly be in excess compared to the surface area (or capacity) of the membrane. Such a process could, however, not be endless. In turn, while being secreted, the lipid droplets are coated with a double membrane and take part of the membrane material away with them. This mechanism of lipid droplet secretion allows the continuity of the milk production process to be maintained. In addition, the secretion mechanism of both hydrophilic and lipophilic substances coordinated in this way enables a stable level of phospholipids in the membrane to be maintained as well as membrane continuity and non-intermittent transport (Fig. 3b). To the author’s knowledge, this observation has not been reported in the literature to date. Considering the above, the mechanism of secretion presented in Fig. 2a seems rather unlikely. In this case, a one-way flux of phospholipids and accumulation of their excess quantities in the apical part of the membrane would occur. A continuous process would be possible on condition that some mechanism would be developed allowing for discharge or removal of phospholipids from the membrane, which has rather not been corroborated by study results.

Milk production is a great challenge for mammary epithelial cells. It requires well-coordinated uptake of systemic building blocks for the production of nutrient-rich milk. The observations discussed above have shed some new light on the organization and coordination of the milk production process in the mammary gland. Considering the flux of phospholipids and their indispensability inside the epithelial cells of the mammary gland, they may be one of the key elements in regulating the secretion of milk components.

### The Milk Fat Globule Size as a Result of Balanced Biosynthesis of Polar and Neutral Lipids

The availability of polar lipids may also greatly affect the size of MFG. Greater availability of phospholipids may enable better dispersion of triacylglycerols in the form of smaller MFG which, in turn, will be reflected in the better availability of fat and better digestion in the organism of the progeny.

By this means, both the flux and availability of phospholipids may determine the ratio of hydrophilic to lipophilic substances in milk, simultaneously enabling their coordination and uninterrupted secretion. The specified volume of transporting vesicles corresponds to the specified amount of the membrane material that will be attached to the apical membrane. This indicates that a specific amount of phospholipids may be used for the secretion of a specified amount (volume) of fat.

Taking into account the changes in milk composition during transition from colostrum to the mature milk, a negative correlation may be observed between the size of fat globules and the content of phospholipids. Using formulas for sphere surface area and volume, the correlation between the number of formed globules with uniform size and the formed surface of milk fat may be expressed with the following formula:

$$ y=4.835\ {x}^{0.333} $$

where: x denotes the number of globules formed and y denotes globule surface area.

According to the formula, assuming a density equal to 1 g/cm^3^, a surface of 48.34 cm^2^ will be achieved once 1000 globules of the same size are produced from 1 g of fat. An increase in the number of produced globules to 2000 will result in surface increase to 60.90 cm^2^. This formula approximately shows the increasing demand for phospholipids (as a monolayer) at an increasing degree of dispersion for a specified amount of triacylglycerols. It indicates that an appropriate amount of phospholipids may be an element controlling fat dispersion degree. Changes in globule size may be determined by the availability of phospholipids (especially in a triple layer), but also have to meet the specific needs of a new-born for phospholipids and triacylglycerols, respectively. This demand may vary throughout the developmental process and, initially, during intensive development of the alimentary and nervous systems a relatively higher supply of phospholipids may be of greater significance. Furthermore, phospholipids contain significant amounts of polyunsaturated fatty acids (PUFA), which are an indispensable component of cellular membranes and are essential for proper brain and retina development in neonates [75]. Zou et al. [76] investigated changes in the lipid composition of human milk during lactation. They showed that fat content increased during lactation and colostrum milk also constituted a higher content in PUFA (ω-6, long chain ω-6 and ω-3) than mature milk, which is of special importance during neonatal development. Most likely, all of these factors do play a role, are correlated with each other and are the result of adaptation in the evolutionary process. However, the contents of phospholipids and triacylglycerols in milk are strictly correlated and are “expressed” by the size of MFG.

Considering the aforementioned limitations, the phospholipids supply to a new-born’s body may be enhanced through so-called “lactosomes”, i.e. small lipid globules free of triacylglycerols, the presence of which has recently been detected in milk [77]. They may also balance the flux of phospholipids by removing their excess from the membrane produced after the supply of liposomes with water soluble components of milk and maintain the continuity of milk production.

The process during which a fat globule is coated with a triple phospholipid membrane may yield some benefits to the progeny ingesting milk. Fat globules coated with the triple phospholipid layer provide significantly higher (ca. threefold) amounts of phospholipids compared to the monolayer. For a developing young organism, the appropriate amount of phospholipids provided with food may be of key significance for the proper development of not only the nervous system, but the organism as a whole.

During intensified production of milk fat in the mammary gland, lipid droplets are also subject to coalescence and fusing into larger droplets in a cell’s interior and, with their sizes increased, they are then secreted outside the cell. As mentioned above, a high degree of lipid droplet dispersion inside cells of the mammary gland, with a concomitant increased intensive production of MFG, requires a high supply of phospholipids. One of the means to reduce the demand for phospholipids with a the simultaneously enhanced production of milk fat involves a reduction of fat dispersion degree via coalescence and the formation of larger fat globules. For such globules, the surface area-to-weight ratio is lower and, resultantly, the demand for phospholipids is reduced. In other words, with a specified “pool” of phospholipids, the increase in fat globule size allows for a significantly greater secretion of milk fat. Thus, the availability of phospholipids may affect the size of intracytoplasmic lipid droplets and may be one of the factors regulating their size.

The above considerations have been confirmed in conducted research. Lopez et al. [78] pointed to a correlation between the content of polar lipids and the surface and size of fat globules. The secretion of small globules leads to a higher MFGM/TAG ratio compared to large fat globules. In turn, Argov et al. [79] demonstrated that the polar lipids-to-triacylglycerols ratio (PL/TAG ratio) decreased along with an increasing fat content of milk. Finally, Wiking et al. [80] demonstrated a correlation between milk fat content and fat globule size and emphasized the availability of membrane material as a limiting factor. These correlations also show the significant role of phospholipids as a factor determining fat content and fat globule size.

### Changes in Fat Globule Size Require Reorganization of the Interface Material

**Fig. 4:**
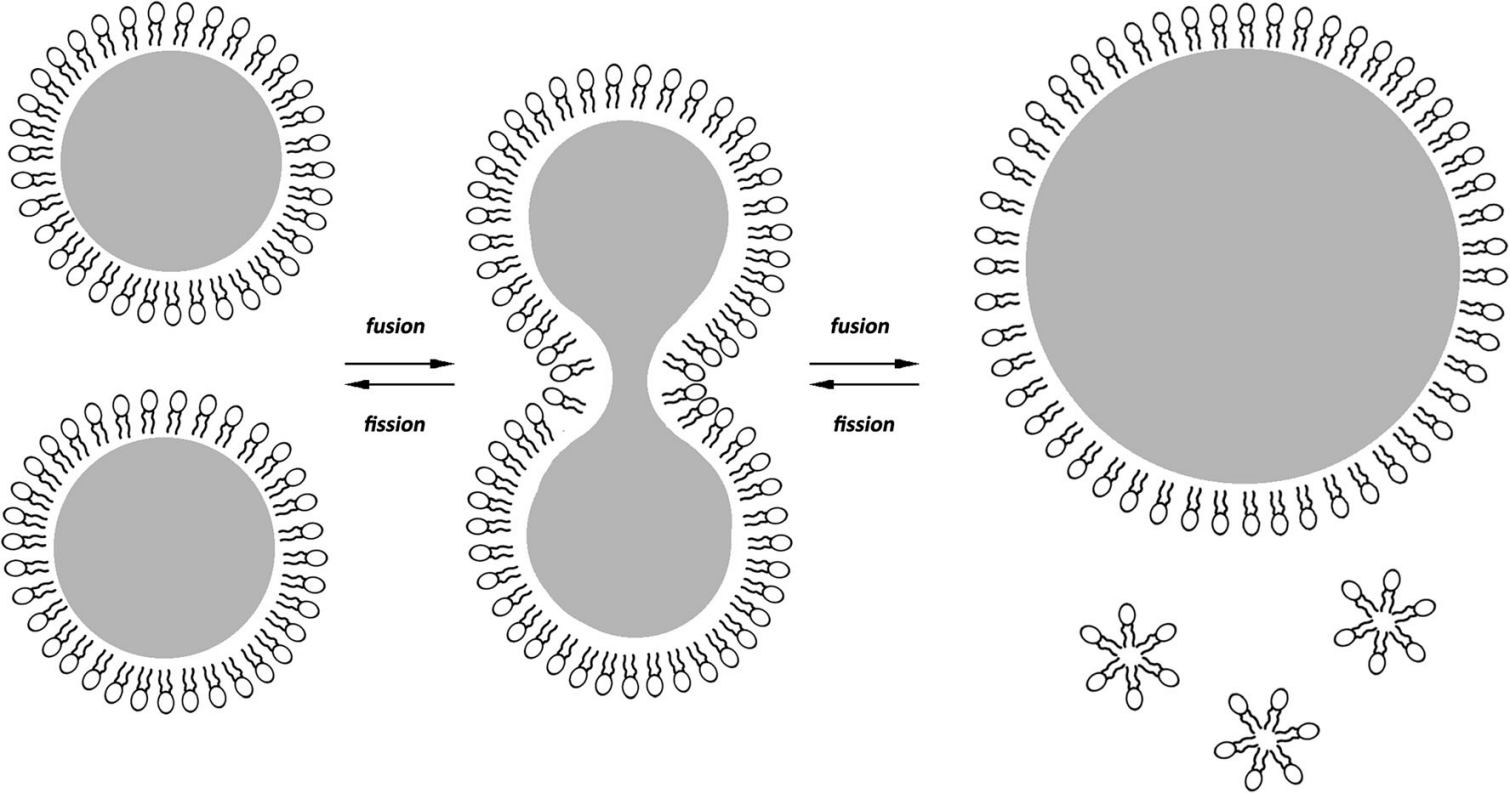
The process of fusion or breakdown of intracytoplasmic lipid droplets requires changes in the content of phospholipids in the membrane. The merging of droplets leads to an excess of phospholipids which have to be removed from the membrane as the volume of the TAG droplet increases. Conversely, the breakdown into smaller droplets requires additional amounts of phospholipids to contain the smaller sized CLD

It is also noteworthy that the process of fusion or breakdown of droplets into smaller ones results in an excess or deficit of phospholipids. It seems advisable, therefore, to consider whether the generated excess or deficit of phospholipids leads to a stable structure characterized by insignificant loosening (or potentially by insignificant concentration) or whether it requires an immediate reorganization of the interface material. However, it seems likely that the process of globule fusion requires removing the excess of phospholipids from the interface in order to ensure stability and durability of a newly-formed system (Fig. 4). By this means, it is also possible that the removal or incorporation of phospholipids (for example with the action of enzymes) into the interface may enforce or initiate the successive processes of either fusion or breakdown of droplets into smaller ones. In this case, not only phospholipid availability but also its metabolism or modifications could be an element enabling lipid droplet size regulation in the cell’s interior. This observation is in agreement with studies showing the role of phosphatidylcholine and phosphatidic acid (PA) in lipid droplet size. The high content of PC is related to small droplets and is believed to prevent coalescence, while only a small amount of PA, a fusogenic lipid, is known to trigger SNARE-dependent and SNARE-independent membrane fusion events leading to increased droplet sizes [62, 81].

A similar mechanism, in which a key role is ascribed to phospholipids, may be applied to regulate the size of intracytoplasmic lipid droplets in other cells. Fat cells (adipocytes) are characterized by considerably larger sizes of intracytoplasmic lipid droplets. It is known that the key role in regulating fat globule size and in management of cell lipids reserves is played by PAT family proteins [82]. However, the mechanism according to which they regulate the access to cell lipids reserves and the mechanism allowing for size regulation of intracytoplasmic lipid droplets remains unclear. Phospholipids may be one of the factors that regulate their size and their effective consumption by interactions with PAT proteins. In this case, with the specified pool of phospholipids and high fat content, increasing the size of lipid droplets may prevent the unnecessary consumption of phospholipids and ensure more effective use of the available intracellular space. In mammary gland cells, the droplets are smaller, which necessitates a greater amount of phospholipids. However, in this case, of greater significance may be the possibility of droplet secretion outside the cell and providing significant amounts of phospholipids to a developing new-born, which is feasible by increasing the degree of milk fat dispersion and the triple phospholipid layer.

## Summary

Through the presentation of characteristic structural traits of milk fat globules and mechanisms linked with their secretion, this work indicates the potential role of phospholipids in this process. To date much work has focused on total lipid composition and how the individual fatty acid composition of milk is altered by maternal diet or targeted gene deletion, but little is known about phospholipid synthesis in mammary gland and its role during lactation. The characteristic properties of phospholipids make them indispensable for the secretion of not only milk fat, but also other milk components. Since these processes are associated with the flux and availability of phospholipids, they have to be strictly coordinated to proceed smoothly. This indicates a strong correlation between secretion of lipid and water-soluble components. The structure formed as a result of these processes, i.e. a milk fat globule, is therefore a result of specified structural limitations inside the cell, the overcoming of which enables the secretion of milk components, although it also needs to reflect the nutritional demands of a developing infant’s body, resulting from evolutionary adaptation.

## Abbreviations

MFG: milk fat globules
MS: mass spectrometry
NMR: nuclear magnetic resonance
MFGM: milk fat globule membrane
MUC: mucin
XDH/XO: xanthine dehydrogenase/oxidase
PAS: periodic acid Schiff
CD: cluster of differentiation
BTN: butyrophilin
EGF: epidermal growth factor
ADPH: adipophilin
FABP: fatty acid binding protein
MHC: major histocompatibility complex
PC: phosphatidylcholine
PE: phosphatidylethanolamine
SM: sphingomyelin
PI: phosphatidylinositol
PS: phosphatidylserine
FA: fatty acids
ER: endoplasmic reticulum
PUFA: polyunsaturated fatty acids
EPA: eicosapentaenoic acid
DHA: docosahexaenoic acid
VLDL: very low density lipoproteins
LPL: lipoprotein lipase
CoA: coenzyme A
SCD: stearoyl-CoA desaturase
GPAT: glycerol phosphate acyltransferase
AGPAT: acylglycerolphosphate acyltransferase
DGAT: diacylglycerol acyltransferase
PA: phosphatidic acid
DAG: diacylglycerol
TAG: triacylglycerol
CK: choline kinase
CTP: cytidine triphosphate
CCT: CTP:phosphocholine cytidylyltransferase
CPT: cholinephosphotransferase
PLA2: phospholipase A2
LPC: lysophosphatidylcholine
LPCAT: lysophosphatidylcholine acyltransferase
ATP: adenosine triphosphate
ADP: adenosine diphosphate
CDP: cytidine diphosphate
CMP: cytidine monophosphate
PP: diphosphate
PRLR: prolactin receptor
SREBP: sterol regulatory element-binding protein
SCAP: SREBP cleavage activating protein
PPAR: peroxisome proliferator-activated receptor
NSF: N-ethylmaleimide-sensitive-factor
SNAP: soluble NSF attachment protein
SNARE: soluble NSF attachment protein receptor
CIDE: cell death-inducing DNA fragmentation factor 45-like effector
Fsp27: fat specific protein of 27 kDa
FITM: fat storage inducing transmembrane
APM: apical plasma membrane
CLD: cytoplasmic lipid droplet
MC: milk components
RER: rough endoplasmic reticulum
MLD: microlipid droplet
N: nucleus
GA: Golgi apparatus
PL: phospholipid
